# ChatGPT: when the artificial intelligence meets standardized patients in clinical training

**DOI:** 10.1186/s12967-023-04314-0

**Published:** 2023-07-06

**Authors:** Xiaoni Liu, Chaoling Wu, Rui Lai, Hai Lin, Yiying Xu, Yuansheng Lin, Weilong Zhang

**Affiliations:** 1 Department of Hematology, Lymphoma Research Center, Peking University Third Hospital, No. 49 North Garden Road, Haidian District, Beijing, 100191 China; 2 Department of Respiratory Medicine, The First Affiliated Hospital of Gannan Medical University, Ganzhou, 341000 China; 3 Department of Respiratory Medicine, Affiliated Hospital of Jiujiang University, Jiujiang, 332000 China; 4 Department of the Respiratory Medicine, The People’s Hospital of Ruijin City, Ruijin, 342500 China; 5 Department of Respiratory and Critical Medicine, Longnan First People’s Hospital, Longnan, 341700 China; 6 Department of Emergency and Critical Care Medicine, Suzhou Hospital, Affiliated Hospital of Medical School, Nanjing University, No. 1 Lijiang Road, Gaoxin District, Suzhou, 215000 China

**Letter to the editor:**

Standardized patients (SP) were an effective and important program to help medical students develop communication skills [1]. Compared with the low-fidelity manikin, the SP group had significantly higher test scores and communication skills [2]. Artificial intelligence (AI) helps guide medical decisions that benefit individuals and populations and provides insights for optimizing various systems such as public health [3]. Since the release of the chat robot ChatGPT, this artificial intelligence technology has clearly had a significant impact on the way humans work [4]. Due to the importance of SP in clinical training and education, we collected 10 patient histories related to clinical training and education using ChatGPT, and evaluated them by senior physicians to verify the accuracy of the information generated by ChatGPT simulating SP.

We provided 10 cases (11 to 15 questions for each case) to ChatGPT (ChatGPT-3.5-turbo, mode) and collected the medical histories of these 10 patients through ChatGPT simulating SP. Based on the answers provided by ChatGPT to each question, then we asked 5 senior physicians to evaluate their accuracy. Senior physicians independently evaluated the accuracy score (0–10 points) of each case and summarized them together.

We used the standardized training exam for resident doctors in Jiangxi Province as an example, and drew the flowchart of SP and ChatGPT simulating SP training respectively (Fig. 1A). Compared with real SP, ChatGPT simulating SP could eliminate the need for complex steps 1–6, meaning that ChatGPT simulating SP did not need to prepare medical records or additional training and could be used directly, saving a lot of time, manpower and resources. After evaluation of 5 senior physicians, out of 10 cases, 2 were rated as 10 points and 6 were rated as 9 points, the remaining 2 cases were rated as 8 points (Fig. 1B). ChatGPT simulating SP played a good role in all stages of consultation. For example, case 4 had 13 questions and an evaluation score of 10 (Fig. 2). Firstly, ChatGPT simulating SP was highly intelligent. When ChatGPT met different cases, it could quickly combine the patient's chief complaints and give appropriate and correct answers. Secondly, ChatGPT simulating SP responses were colloquial. When ChatGPT was responding to a doctor’s query, it answered the questions in a tone close to that of the patient, such as in questions 2 and 6. Thirdly, ChatGPT simulating SP’s responses were vivid and accurate. When ChatGPT described symptoms, it would use vivid words and answer accurately, as demonstrated in question 7. However, ChatGPT simulating SP’s responses were mechanical and rigid occasionally when we asked more than one question at a time, such as in cases 9 and 12. The descriptions of symptoms reached the level of our clinical skills training and SP simulation in the test station. In general, ChatGPT can simulate the complete medical record interrogation mode without disconnection and can be applied to the SP interrogation mode.

**Fig. 1:**
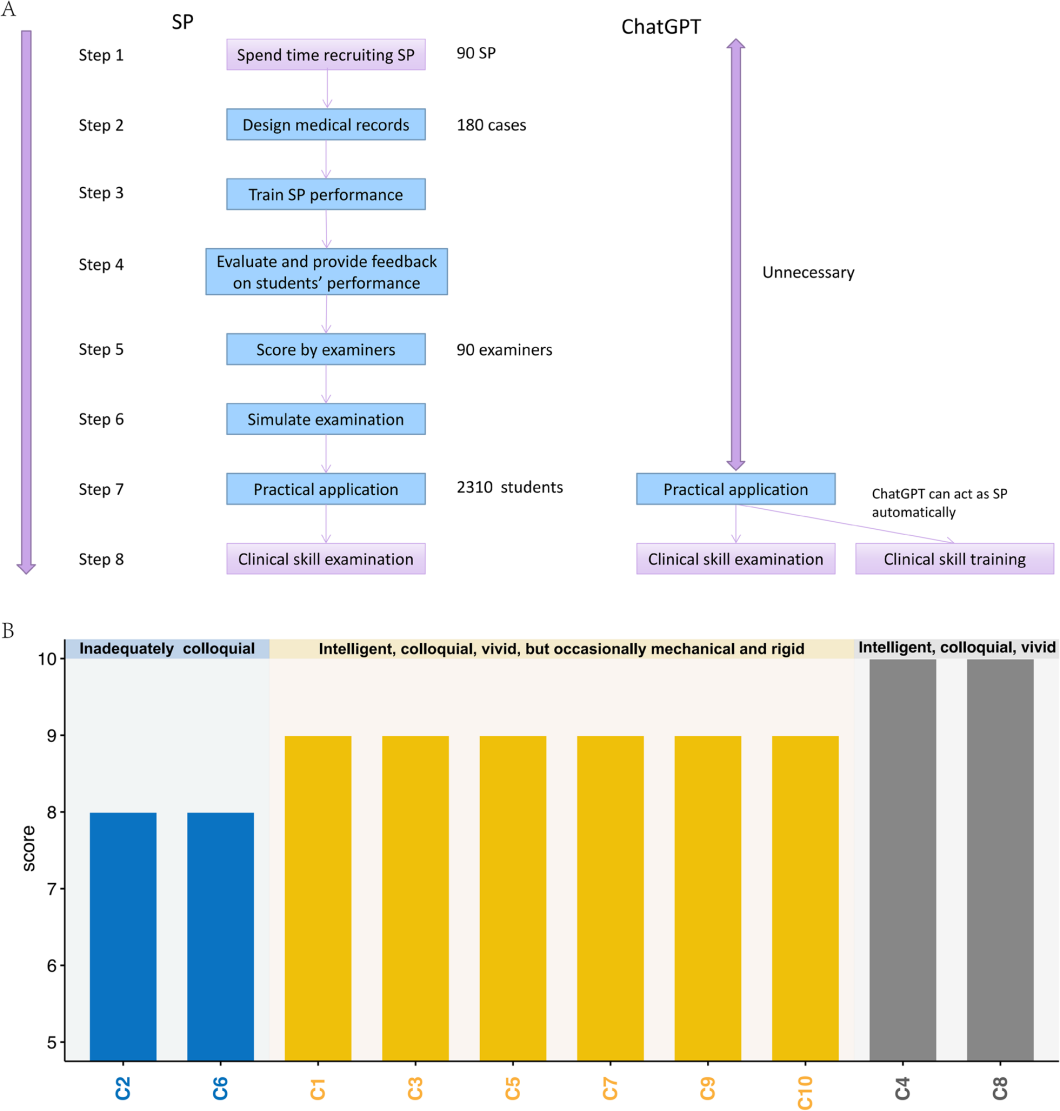
**A**. Flow chart of SP and ChatGPT simulating SP training. **B**. Evaluation scores and characteristics of 10 cases

**Fig. 2:**
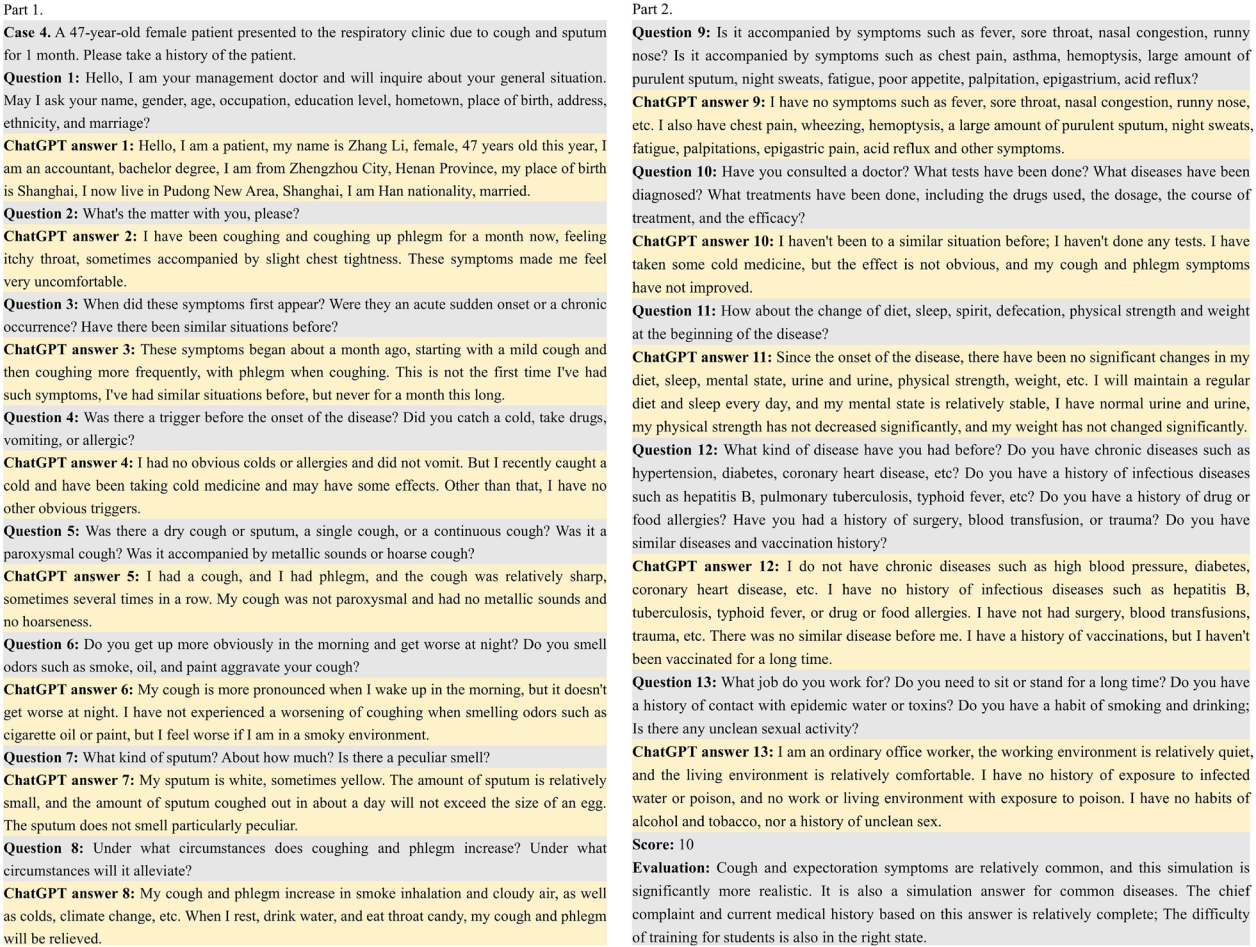
ChatGPT communicated with physicians as SP, and its responses were evaluated by senior physicians. Gray represents the questions we asked, and light yellow indicates the answers responded by ChatGPT

In the past few years, ChatGPT has already impacted and made progress in many fields, such as healthcare and education [5]. Nevertheless, ChatGPT has not been reported on simulating SP. In our study, ChatGPT had several advantages in simulating SP. ChatGPT simulating SP was able to combine themes and responded intelligently, colloquially, vividly and accurately, enabling it to play various patient roles. However, there were also some drawbacks, such as ChatGPT simulation of SP's responses being mechanical and rigid occasionally.

Therefore, our results showed that ChatGPT simulating SP could assist in clinical training and education, thereby more effectively guiding doctors' clinical skills, optimizing the education system, and improving medical skills. Meanwhile, it could alleviate the problem of human resource shortage when training real SP. Of course, the problems and limitations of ChatGPT simulating SP required us to maintain criticism, continuously improve and optimize during the use process.

In the future, we look forward to ChatGPT being applied as an SP in our medical education and training. Of course, to ensure the optimal integration of artificial intelligence-based learning tools in medical education, further research and evaluation are still needed.

## Data Availability

Not applicable.

## Abbreviation

SP: Standardized patients
